# Aqua Regia-Free Removal of Cr-Pt Hard Masks Using Thin Ag or Au Sacrificial Layers for High-Fidelity LiTaO_3_ Metasurfaces

**DOI:** 10.3390/nano16010059

**Published:** 2025-12-31

**Authors:** Zhuoqun Wang, Yufeng Zang, Yuechen Jia, Ning Lu

**Affiliations:** 1 School of Physics, State Key Laboratory of Crystal Materials, Shandong University, Jinan 250100, China; 2 School of Microelectronics and Communication Engineering, Chongqing University, Chongqing 400044, China; zangyufeng@stu.cqu.edu.cn; 3 School of Chemistry and Chemical Engineering, Shandong University, Jinan 250100, China

**Keywords:** LiTaO_3_, hard masks, sacrificial layers

## Abstract

For the method of focused ion beam (FIB) milling to fabricate lithium tantalate (LiTaO_3_) metasurfaces, the use of a Cr-Pt mask can enhance imaging contrast and enable superior drift correction. However, removing the Pt component necessitates the volatile and toxic etchant aqua regia, presenting considerable safety risks. This work introduces a novel lift-off strategy that incorporates thin Ag or Au sacrificial layers (≤30 nm) between the LiTaO_3_ substrate and Cr-Pt mask. Systematic evaluation identifies Ag or Au as optimal candidates due to their high sputtering yield for efficient FIB patterning and compatibility with a low-toxicity KI + I_2_ etchant. Experiments showed complete mask removal within 60 s while preserving structural fidelity: atomic force microscopy (AFM) results reveal a surface roughness comparable to conventional aqua regia processing, and scanning microscope (SEM) imaging confirms intact sidewall angles (10–11°). The second-harmonic generation (SHG) measurements reveal comparable optical performance upon the introduction of Ag or Au sacrificial layers. This approach eliminates hazardous etchant and maintains process precision, offering a scalable and safer fabrication route for LiTaO_3_-based photonic devices.

## 1. Introduction

Metasurfaces, comprising periodic arrays with specific dimensions, offer exceptional control over fundamental light parameters (amplitude, phase, frequency, refractive index) as well as diverse optical properties (wavefront shaping, polarization conversion, energy control) [1,2,3]. LiTaO_3_ with its excellent physical and optical properties—such as physico-chemical stability and a broad optical transmission range—is an ideal material for fabricating optical metasurfaces [4,5,6]. However, the high lithium content in LiTaO_3_ presents a challenge: conventional processing techniques (e.g., reactive ion etching) for LiTaO_3_ counterpart accumulate substantial lithium fluoride (LiF) particles [7,8]. These particles degrade optical performance and are incompatible with the stringent surface smoothness requirements essential for metasurfaces.

Focused ion beam (FIB) milling, being primarily a physical sputtering process via ion bombardment with minimal chemical reactions, readily achieves smooth sidewalls [9]. These characteristics position FIB as a promising technique for micro-/nano-fabrication of LiTaO_3_ devices [10,11,12]. Previous research demonstrated that a chromium (Cr) metallic mask effectively mitigates the surface damage induced by ion irradiation during FIB milling, owing to Cr’s exceptionally low sputtering yield (the average number of atoms ejected per incident ion) [5,13,14]. However, Cr exhibits a high secondary electron yield under Ga^+^ ion bombardment, leading to a severe charging effect within the milling region [15,16]. This effect significantly degrades image contrast, hindering clear visualization and identification of the metasurface pattern during fabrication. Further, it impairs the critical drift correction process based on pattern recognition, ultimately compromising fabrication quality. Since the corresponding secondary electron yield of platinum (Pt) is lower than that of Cr, incorporating Pt into the Cr mask (e.g., Cr-Pt alloy) could reduce the secondary electron yield, which effectively improves the contrast of metasurfaces [16]. In further process, removing Cr and Pt masks necessitates the etchants ceric ammonium nitrate (containing HNO_3_ and (NH_4_)_2_Ce(NO_3_)_6_) and aqua regia (a mixture of HNO_3_ and HCl), respectively [17,18]. Since Pt is insensitive to ceric ammonium nitrate, aqua regia is inevitable for Pt removal. Unfortunately, aqua regia poses significant hazards, presenting considerable safety risks during experimental procedures. Therefore, implementing a low-toxicity mask removal method without compromising fabrication quality would substantially enhance safety in both research and production settings.

Established lift-off processes combining traditional photoresists with metallic hard masks are widely used in fabricating lithium niobate (LiNbO_3_) photonic chips and metallic arrays [19,20]. Specially, this method involves spin-coating photoresist onto the substrate, patterning it via conventional exposure and development, depositing the target metallic film, and then rinsing the underlying photoresist in a stripper. As a result, it dissolves this sacrificial layer, causing the overlying metal to lift off from the substrate, leaving behind the metal pattern directly contacting the substrate. This demonstrates that inserting a sacrificial layer (such as photoresist) between the metallic mask and substrate enables safe removal of the top metallic mask (using solvents). However, if photoresist is inserted between the Cr-Pt mask and the LiTaO_3_ substrate, its typical spin-coated thickness (hundreds of nanometers) significantly exceeds that of the upper Cr-Pt mask (tens of nanometers). Consequently, during metasurface fabrication, ion beam divergence within the thick photoresist layer will compromise fabrication precision [21]. Therefore, alternatively sacrificial layer materials that enable safe (viz., with a non-toxic etchant) removal while maintaining minimal thickness must be employed.

Building on this foundational motivation, we introduce a novel strategy designed to circumvent the safety hazards inherently associated with utilizing toxic and volatile etchants necessary for the removal of Cr-Pt mask. Instead of directly etching the Pt-containing mask itself, this work proposes the integration of a thin (≤30 nm) sacrificial layer (Ag or Au) between the LiTaO_3_ substrate and Cr-Pt mask. This specific selection of sacrificial layer material is critically guided by two key attributes: (i) relatively high sputtering yield, thereby minimizing potential conflict with the Cr-Pt mask on the role of protective function under ion milling; and (ii) rapid dissolution kinetics in a low-toxicity of KI + I_2_ solution, enabling easy and safe removal. Collectively, this innovative approach eliminates the critical dependence on hazardous aqua regia while simultaneously preserving crucial lithographic fidelity essential for device performance, enabling safe high-precision fabrication of LiTaO_3_ photonic devices.

## 2. Experimental Section

Layers of Ag, Au and co-sputtered Cr-Pt as well as Ag/Cr-Pt and Au/Cr-Pt bilayers were deposited on LiTaO_3_ substrates (with LiTaO_3_/SiO_2_/Si structure) by DC magnetron sputtering system (Model B22-068, SKY Technology Development Co., Ltd., Shenyang, Liaoning, China). Before deposition, the substrates were cleaned in acetone via ultrasonication for 5 min, followed by rinsing in deionized (DI) water and drying with N_2_ blow. During deposition, all layers were sputtered with a deposition rate of 0.1–0.2 nm/s in the Ar atmosphere with a pressure of 2.5 Pa. By adjusting the power and sputtering time, the final thickness of every layer was controlled at approximate 30 nm. After deposition, all samples were treated by annealing in Ar atmosphere at 200 °C for 9 h.

After that, the metasurface structure was prepared through FIB system (Helios 5 CX, Thermo Fisher Scientific Inc., Waltham, MA, USA). During irradiation, the acceleration voltage and beam current of Ga^+^ were set to 30 kV and 0.23 nA, respectively. The geometric size of metasurface is set as 20 × 20 μm, with a period of 1.3 × 1.3 μm. The milling template (see Figure S1) was designed externally and imported as a bitmap, meaning the entire structure was milled in a single, holistic process without involving serial or parallel milling modes. The FIB scanning was performed in a serpentine pattern, with the scanning direction alternated every 100 s (top-to-bottom, bottom-to-top, left-to-right, right-to-left). Based on the parameters, using a beam current of 0.23 nA over an etching area of 100 μm^2^ (white area of template), the calculated dose is approximate 1.4 × 10^7^ ions/μm^−2^. Parameters such as defocus, blur, and interaction diameter were all set to 0 nm. The dwell time for the FIB was 1.0 μs. After about 600 s ion beam irradiation, a milling depth of 200 nm is achieved. To monitor the drift condition, ion induced secondary electron (iSE) imaging was performed at 100 s interval. The profile of metasurface structure and energy-dispersive X-ray spectroscopy (EDS) analysis were examined by SEM (the same equipment of Helios 5 CX, Thermo Fisher Scientific).

Then, the Cr-Pt layer was removed in aqua regia etchant (concentrated HNO_3_:HCl, 1:3 *v*/*v*). The Ag, Au, Ag/Cr-Pt and Au/Cr-Pt layers were removed in the etchant KI + I_2_ (KI:I_2_:DI at 20 g:6 g:100 mL). After that, all the samples were rinsed in DI water and dried with N_2_ blow. Additionally, aqua regia etching is conducted at room temperature in a fume hood, while the KI + I_2_ solution etching is performed under ambient conditions. Afterwards the morphology of metasurface was characterized by atomic force microscopy (AFM, Bioscope Resolve, Bruker Corp., Billerica, MA, USA). After carbon coating, the profile of metasurface structure and corresponding EDS analysis were examined by SEM.

## 3. Results and Discussion

### 3.1. Concept Description

**Figure 1:**
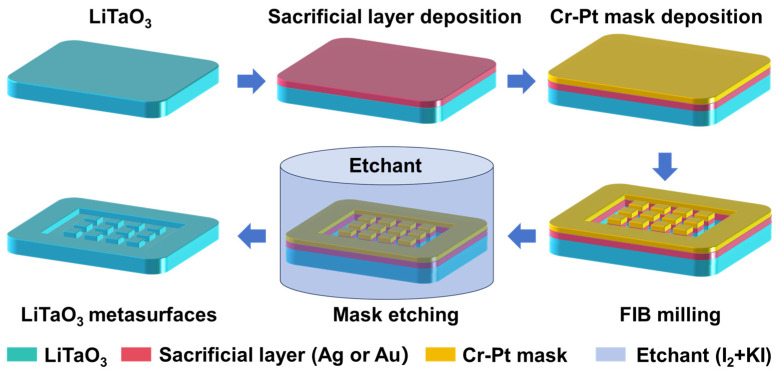
Schematic diagram of the processing flow to remove Cr-Pt co-sputtered mask associated with sacrificial layer.

The concept for safely removing the Cr-Pt mask is illustrated in Figure 1. Initially, a readily removable material, termed as “sacrificial layer”, is deposited on the LiTaO_3_ substrate. Subsequently, the Cr-Pt co-sputtered mask is deposited on the top of sacrificial layer, followed by an annealing process to remove residual stress in metallic layers [22]. Then, the desired metasurface structures on the LiTaO_3_ substrate is fabricated through FIB milling. Finally, immersion in the appropriate etchant selectively dissolves the sacrificial layer. Complete dissolution of this layer detaches the Cr-Pt mask, exposing the final etched metasurface structure.

This process imposes the following requirements on the sacrificial layer: the material must exhibit a high sputtering yield to ensure it is being easily sputtered without significantly interfering with the Cr-Pt mask’s protective function under ion irradiation, thereby simplifying experimental design; while its corresponding etchant should possess both low toxicity and volatility to minimize health hazards and relax containment requirements, enhancing overall applicability.

### 3.2. Mask Materials Survey

We systematically evaluate key physico-chemical properties of common metallic mask materials to assess their viability as sacrificial layers, as shown in Table 1. Criteria include the sputtering yield under 30 keV Ga^+^ bombardment, compatible etchants, and etchant toxicity/volatility [23,24,25]. Among Cr, Ni, Al, Ti, Au, W, Mo, TiN, Co, and Ag, the materials with a sputtering yield comparable to or exceeding Cr (4.6) are Ni (7.9), Au (17.0), W (7.4), Mo (5.5), Co (7.6) and Ag (13.2). In fact, Ni, W, Mo and Co are rejected due to the high toxicity and volatility of their HNO_3_-based etchants. Ag and Au, etchable by a KI + I_2_ solution, present mitigated safety concerns despite elemental iodine’s irritant properties. For example, the equilibrium reaction KI + I_2_ ⇌ KI_3_ favors triiodide ion (I_3_^−^) formation, which substantially reduces free I_2_ volatility. Additionally, optimizing the KI concentration (e.g., excess KI) can further minimize hazards by driving I_3_^−^ formation [26,27]. Thus, both Ag and Au represent viable and comparatively safe alternatives for sacrificial layer application. It should be emphasized that the data in Table 1 are not original results but are intended solely to facilitate comprehension.

### 3.3. Experimental Validation

**Figure 2:**
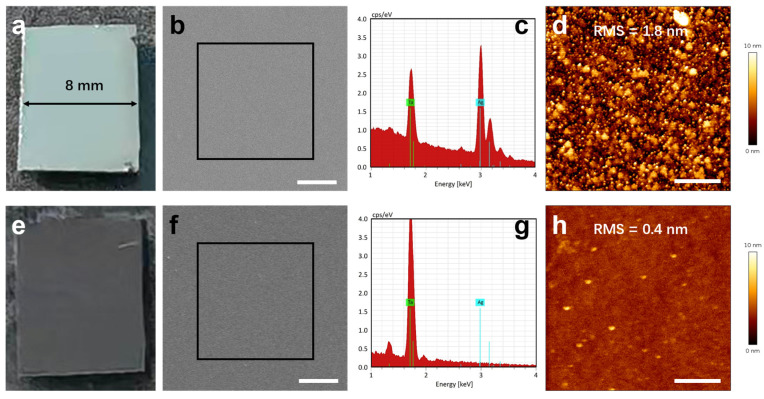
Morphological evolution of Ag-coated sample before and after etching. (**a**) Optical image before etching. (**b**) Corresponding SEM image. Scale bar: 1 µm. (**c**) EDS spectrum taken from the boxed region in (**b**). (**d**) Corresponding AFM image before etching. Scale bar: 500 nm. (**e**) Optical image after etching. (**f**) Corresponding SEM image. Prior to characterization, the sample is coated with carbon to enhance the surface conductivity of the insulating LiTaO_3_ substrate. Scale bar: 1 µm. (**g**) EDS spectrum of the boxed region in (**f**). (**h**) Corresponding AFM image after etching. Scale bar: 500 nm.

The etch rates of Ag and Au in KI + I_2_ solution were semi-quantitatively evaluated by using optical monitoring. Thin films (thickness of ~30 nm) of each metallic layer were deposited on LiTaO_3_ substrates, and then etched under ambient conditions. The Ag film transitions from opaquely metallic reflection (see Figure 2a) to dark gray color (revealing the underlying Si, see Figure 2e) within approximate 20 s, corresponding to an estimated etch rate of 1.5 nm/s. Additionally, SEM-EDS analysis shows that the characteristic X-ray signal of Ag (e.g., La at 2.98 keV) is clearly present before etching (Figure 2c) but is no longer detectable afterward (Figure 2g), indicating the removal of the Ag film. It should be noted that the SEM image after etching (Figure 2f) shows a likely greater surface roughness compared to it before etching (Figure 2b). This apparent increase may be attributed to the carbon coating applied prior to post-etch SEM imaging to enhance the conductivity of the insulating LiTaO_3_ substrate—a step that can itself modify surface topography (see Figure S2). This assumption is further verified by AFM measurements, which do not require conductive layer coating. The AFM results demonstrate that the as-deposited Ag film has a relatively high surface roughness (RMS = 1.8 nm, as shown in Figure 2d), whereas the surface becomes significantly smoother after etching (RMS = 0.4 nm, as shown in Figure 2h).

In addition, the Au film loses its metallic reflectivity and exhibits substantial fading within just 5 s, suggesting a significantly higher etch rate exceeding 6 nm/s (see Figure S3a,e). The results indicate that the characteristic X-ray signal of Au (e.g., Ma at 2.12 keV) is intense before etching (Figure S3c) but becomes nearly undetectable afterward (Figure S3g), indicating the removal of the Au layer. Owing to the carbon coating applied for conductivity enhancement, the post-etch SEM image (Figure S3f) appears rougher than that before etching (Figure S3b). AFM measurements further verify that the as-deposited Au film exhibits relatively high roughness (RMS = 0.6 nm, as shown in Figure S3d), which decreases slightly after etching (RMS = 0.4 nm, as shown in Figure S3h).

These findings demonstrate that both the Ag and Au layers are etched away by the KI + I_2_ solution, exposing the underlying LiTaO_3_ substrate, which itself shows relatively low roughness. Although this visual monitoring method cannot provide precise kinetic data, it effectively establishes the order-of-magnitude of etch rate of those two metals.

Subsequently, metasurfaces were fabricated by employing Ag or Au as sacrificial layers to validate the mask removal process. The Ag or Au layers were firstly deposited on LiTaO_3_ substrates via magnetron sputtering, followed by the co-deposition of Cr-Pt mask. After thermal annealing, the metasurface arrays were patterned using FIB milling. For comparative assessment of the mask removal efficacy, a control sample with only the Cr-Pt mask (without any sacrificial layer) was fabricated using an identical process flow. The metallic layers were removed under distinct conditions: the control sample undergoes aqua regia etching for 15 min, whereas the samples with Ag or Au sacrificial layers are etched in KI + I_2_ solution for 1 min.

**Figure 3:**
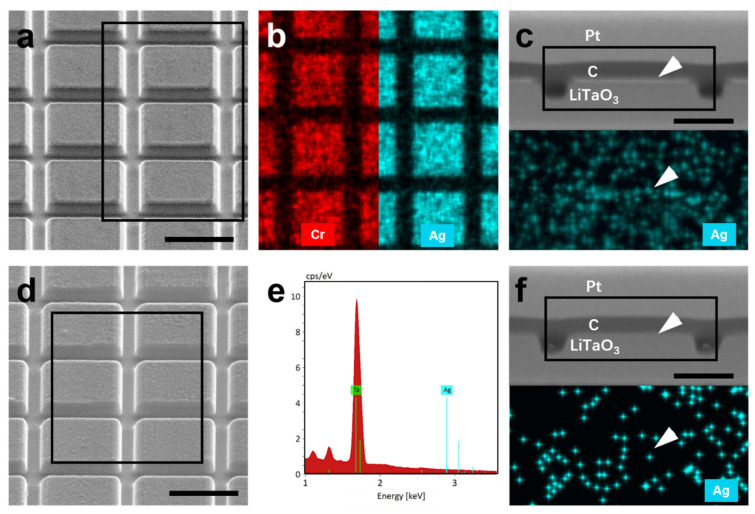
Compositional evolution of the sample with Ag/Cr-Pt mask before and after etching. (**a**) SEM image of the sample before etching, acquired at a 52° tilt. Scale bar: 1 μm. (**b**) Corresponding elemental maps of Cr and Ag from the boxed region in (**a**). (**c**) Cross-section SEM image and Ag elemental map corresponding to the boxed region. Scale bar: 500 nm. (**d**) SEM image after etching, taken at a 52° tilt. Prior to imaging, the sample is coated with carbon to enhance conductivity. Scale bar: 1 μm. (**e**) EDS spectrum collected from the boxed region in (**d**). (**f**) Cross-section SEM image and corresponding Ag elemental map from the boxed region. Scale bar: 500 nm. White arrows indicate the surface position of the metasurface units in (**c**,**f**).

To evaluate the etching effect, morphological and compositional analyses were performed on the samples before and after etching. Taking the sample with Ag/Cr-Pt mask as an example, the SEM observation (acquired at a 52° tilt) reveals a relatively smooth surface prior to etching (see Figure 3a). Corresponding EDS mapping reveals distinct X-ray signals for Cr and Ag distributed across the metasurface units (see Figure 3b), confirming that both the Ag and Cr-Pt layers remain intact after the etching step. Cross-section SEM and EDS analysis further show bright contrast at the surface of the metasurface units, along with a strong Ag signal (see Figure 3c), again verifying that the mask layer is retained. After etching, the SEM imaging indicates an increase in surface roughness (see Figure 3d), which is likely attributable to carbon coating as discussed above. The corresponding EDS spectrum shows that the Ag signal (e.g., La at 2.98 keV) becomes nearly undetectable, as shown in Figure 3e. Moreover, cross-section observation reveals that the previously bright contrast on top of the metasurface units has disappeared, and no Ag signal is detected in this region (see Figure 3f), confirming the removal of the Ag/Cr-Pt mask after etching.

Similar analyses were also performed on the Cr-Pt and Au/Cr-Pt samples before and after etching, as shown in Figures S4 and S5. EDS results show distinct X-ray signals from the mask-layer elements before etching, which are no longer detectable afterward, confirming the removal of those masks. The sidewall profiles were also analyzed using the above cross-section imaging. However, environmental vibration-induced sample drift leads to asymmetric sidewalls in the metasurface units, preventing a meaningful comparison of profiles across the three sample types (see Figure 3, Figures S4 and S5). Furthermore, the overall morphology of all three samples was examined by optical microscopy before and after etching (see Figure S6). However, at a high magnification of 500×, no color difference among the three sample types could be discerned, nor were any visible changes observed after etching under these imaging conditions. Additionally, samples coated solely with Ag or Au layers were subjected to FIB fabrication, as demonstrated in Figure S7. It is evident that the structural integrity of these layers collapse under ion milling, which can be attributed to the relatively high sputtering yields of Ag and Au (see Table 1).

**Figure 4:**
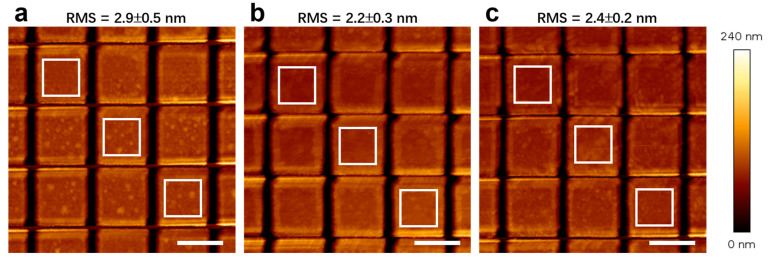
AFM images of LiTaO_3_ metasurfaces after the removal of (**a**) Cr-Pt mask (control sample), (**b**) Ag/Cr-Pt mask and (**c**) Au/Cr-Pt mask. The surface roughness is measured from the white boxed regions. All scale-bars measure 1 μm.

After rinsing with deionized water and drying with N_2_ blow (without carbon coating), the surface morphology was characterized by AFM. As shown in Figure 4, the surface roughness of the etched metasurfaces was measured to be 2.9 ± 0.5 nm, 2.2 ± 0.3 nm, 2.4 ± 0.2 nm for the samples with Cr–Pt mask, Ag/Cr–Pt mask, Au/Cr–Pt mask, respectively, indicating generally comparable roughness across those three sample types, with slightly lower values observed for the Ag- and Au-containing samples. In the Cr–Pt maksed sample, localized particulate features contributed to the higher roughness. Although SEM-EDS results confirm the removal of the Cr–Pt mask (see Figure S4), the higher roughness likely stems from the irradiation damage to the underlying LiTaO_3_ substrate. This can be attributed to the thinner mask in the Cr–Pt sample, whereas the added Ag and Au sacrificial layers in the other two systems provide greater overall thickness and thus enhanced substrate protection during ion milling. Collectively, the results above demonstrate that the sacrificial layer method effectively eliminates the need for toxic and volatile aqua regia in the removal of Pt-containing masks. Notably, the AFM topographic characterization was conducted in tapping mode, where the probe is mounted on a cantilever. During scanning, the bending of the cantilever introduces an anisotropic deflection. This results in inconsistent probing depths along the X and Y axes, particularly within the grating grooves (cause more pronounced bending), leading to variations in imaging fidelity (see Figure 4).

An attempt was also made to characterize the pre-etch metasurface topography using AFM. However, the AFM measurements indicate a loss of mask integrity (see Figure S8a–c), manifesting as anomalous height protrusions along the unit peripheries. Follow-up SEM analyses corroborated this finding by revealing corresponding structure alterations in the masks at the unit edges (see Figure S8d–f), which are different from the smooth surface observed right after FIB milling (see Figure 3, Figures S4 and S5). The anomalies are hypothesized to be artifacts induced by the AFM tip during tapping-mode operation, potentially disrupting the mask-substrate adhesion and leading to localized lifting. A detailed understanding of this mechanism awaits further study.

### 3.4. Fabrication Quality Verification

**Figure 5:**
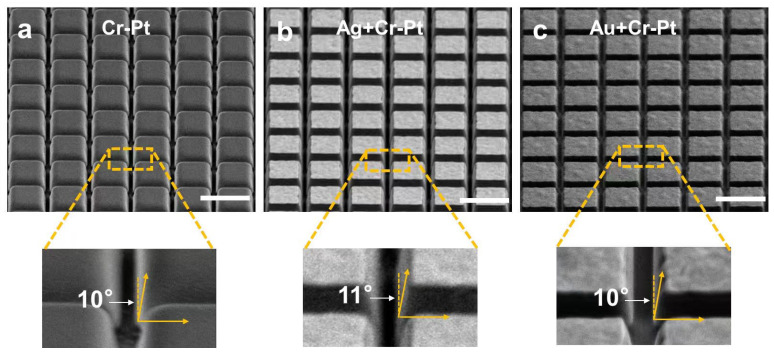
SEM imaging of the LiTaO_3_ metasurface coated with various materials: (**a**) Cr-Pt mask, (**b**) Ag sacrificial layer plus Cr-Pt mask and (**c**) Au sacrificial layer plus Cr-Pt mask. The samples are tilted to 52° during lateral observation. The mask layers were intentionally retained on the sample surface prior to SEM imaging. All scale-bars measure 1.5 μm.

To evaluate the potential impact of mask material modification on FIB milling fidelity, the topographic feature (e.g., sidewall angle, defined by the sidewall tilt relative to normal vector) is characterized through SEM imaging. It should be noted that the mask layers were intentionally retained on the sample surface to mitigate charging effects. During observation, the sample is tilted to 52° to enable lateral observation of the metasurface structure, as shown in Figure 5. Comparative analysis reveals that both the control sample (fabricated with a Cr-Pt mask) and the modified samples (incorporating Ag or Au sacrificial layers) achieve equivalent metasurface sidewall angles of approximate 10–11°. This confirms that the alternative fabrication approaches induce no significant variation in milling profile. Furthermore, the sidewalls maintain high smoothness across all samples, with no observable microscale roughness or irregular topography. These results demonstrate that the incorporation of Ag or Au sacrificial layers has a negligible impact on the milling quality of LiTaO_3_ metasurfaces, thereby preserving the critical sidewall steepness and surface uniformity required for high-performance devices.

Additionally, the sample coated with Au/Cr-Pt layers was FIB milled to different depths to examine morphological evolution, as revealed in Figure S9. It can be observed that with the increase in milling depth, the sidewall profile of metasurface structures remains largely unchanged, indicating that greater milling depth does not significantly alter the sidewall morphology.

**Figure 6:**
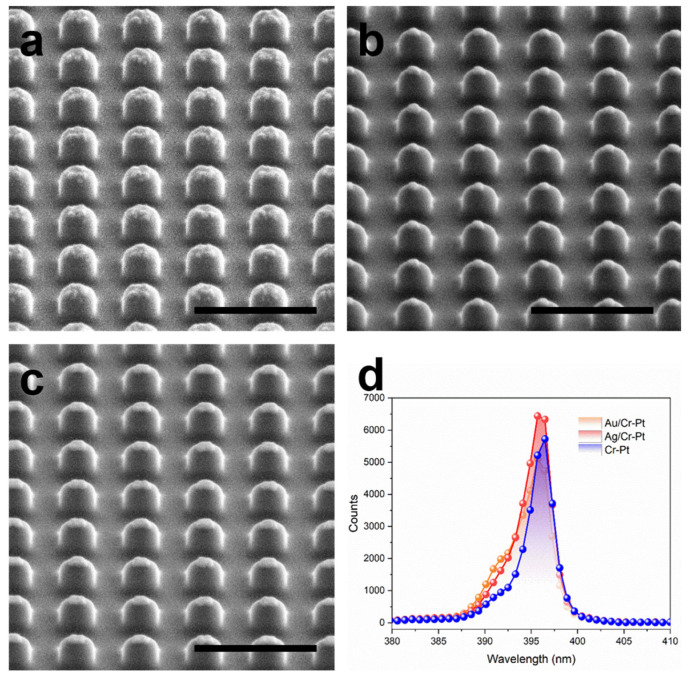
Optical performance of LiTaO_3_ metasurfaces. (**a**–**c**) LiTaO_3_ metasurfaces patternning with Cr-Pt, Ag + Cr-Pt and Au + Cr-Pt masks, with period of 540 nm, width of 300 nm and height of about 200 nm. All scale bars measure 1 μm. (**d**) SHG intensity among those three metasurfaces.

Using the fabrication method established in this study, three LiTaO_3_ metasurfaces were patterned with Cr-Pt, Ag/Cr-Pt and Au/Cr-Pt masks (Figure 6a–c) under consistent processing conditions. To evaluate their second-harmonic generation (SHG) response, reflection-mode SHG measurements are conducted with a home-built micro-spectroscopic characterization setup integrated with a Ti:sapphire femtosecond laser (TiF DP, Avesta Ltd., Moscow, Russia) as the excitation source. The laser provides 60 fs pulses at a repetition rate of 90 MHz and is tunable across 760–820 nm. The polarization of the incident beam is adjusted via a half-wave plate before being focused onto the sample through a 20× microscope objective (N.A. = 0.45), resulting in a spot size of approximately 5–10 μm. The reflected SHG signal is collected by the same objective, filtered with a bandpass filter, and subsequently analyzed using a spectrometer (NOVA 2S-EX, Ideaoptics Instruments Co., Shanghai, China). The measurements in Figure 6d reveal no notable difference in SHG intensity among the three metasurfaces under the same pumping condition, indicating that their nonlinear optical performances are similar.

### 3.5. Discussion

For metasurface unit cells sized at 1.3 × 1.3 µm, complete removal of the sacrificial layer within 60 s in KI + I_2_ solution necessitates a minimum etch rate of approximate 11 nm/s. The experimentally determined dissolution rates (>6 nm/s for Au and ~1.5 nm/s for Ag, see Section 3.3) indicate that only Au could theoretically meet this requirement. Nevertheless, successful removal is achieved with both metals. This discrepancy reveals a significant electrochemical acceleration mechanism specifically enhancing Ag dissolution.

**Figure 7:**
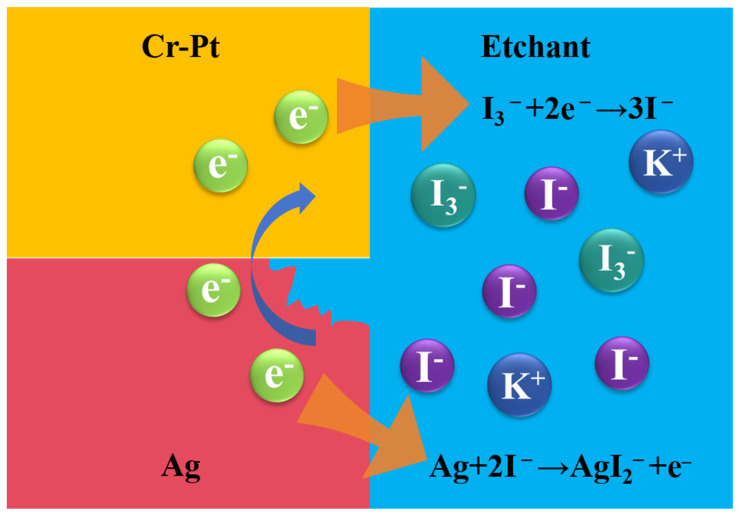
Schematic of the galvanic configuration formed between the Ag sacrificial layer (anode) and the Cr-Pt hard mask (cathode).

This phenomenon is likely attributed to the galvanic corrosion, driven by the electrochemical potential difference between the Ag sacrificial layer (standard electrode potential: +0.80 V vs. SHE) and the Cr-Pt mask (dominated by Pt at +1.20 V vs. SHE) [28,29]. The observed rapid Ag dissolution in KI + I_2_ solution can be mechanistically described by a redox reaction analogous to that of Au: 2Ag + I_3_^−^ + I^−^ → 2AgI_2_^−^. In this galvanic couple as described in Figure 7, the electrochemically stable Pt acts as an efficient cathode, facilitating the reduction reaction: I_3_^−^ + 2e^−^ → 3I^−^. Concurrently, the anodic dissolution of Ag is conducted: Ag + 2I^−^ → AgI_2_^−^ + e^−^. Consequently, such synergistic cycle drives sustained Ag oxidation beyond its intrinsic dissolution kinetics while simultaneously regenerating the etchant (iodide ions) at the Pt cathode interface. As a result, this configuration further enables the highly efficient and complete lift-off of the Cr-Pt mask.

**Figure 8:**
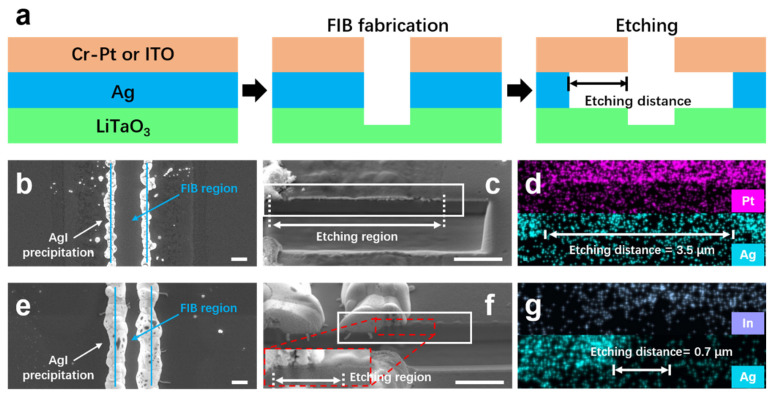
Verification of the galvanic effect between Ag and Cr–Pt. (**a**) Schematic of the corrosion distance measurement. An Ag sacrificial layer is firstly deposited on a LiTaO_3_ substrate, followed by the deposition of either a Cr–Pt layer or an indium tin oxide (ITO) layer. Then they are processed by FIB milling and subsequently corroded in a diluted KI–I_2_ solution (KI: I2: DI at 20 g: 6 g: 500 mL). Finally, the corrosion distance of the Ag layer is measured. (**b**,**c**) Plan-view and cross-section SEM images of the corroded Ag/Cr–Pt sample. (**d**) EDS elemental maps for Pt and Ag corresponding to the boxed region in (**c**). (**e**,**f**) Plan-view and cross-section SEM images of the corroded Ag/ITO sample. (**g**) EDS elemental maps for Pt and Ag corresponding to the boxed region in (**f**). All scale-bars measure 1 μm.

To verify this galvanic configuration hypothesis, samples with Ag/Cr-Pt and Ag/ITO masks were prepared for comparative experiments. The indium tin oxide (ITO), being an oxide ceramic rather than a metal, does not form a galvanic couple with Ag, thus making it suitable to be served as a reference. The experimental procedure is as follows (see Figure 8a): after Ag/Cr-Pt and Ag/ITO samples preparation, the sample cross-sections will be exposed via FIB milling; both samples are then immersed in a diluted KI + I_2_ etchant (KI:I_2_:DI at 20 g: 6 g: 500 mL, with extended etching time of 180 s to improve temporal resolution) for an identical duration, followed by rinsing in ethanol for cleaning; SEM characterization is performed to determine the lateral corrosion length of the Ag layer.

Plan-view and cross-section SEM images reveal obvious corrosion of the Ag layer in the Ag/Cr-Pt sample (see Figure 8b,c), characterized by a distinct change in contrast, loss of mask integrity, and apparent delamination from the substrate. Corresponding EDS results confirm a pronounced decrease in Ag signal within the corroded region, as shown in Figure 8d. Based on the SEM images and EDS mapping results, the measured lateral corrosion depth is approximate 3.5 µm. In contrast, plan-view SEM of the Ag/ITO sample shows ambiguous corrosion of the Ag layer, as shown in Figure 8e. While, a magnified view in the cross-sectional SEM image indicates corrosion (see Figure 8f), evidenced by a darkening contrast at the original Ag layer location. Corresponding EDS analysis also verified a weakened Ag signal in this region, as illustrated in Figure 8g. Accordingly, the lateral corrosion depth here is determined as ~0.7 µm. It should be noted that although the samples are rinsed in ethanol, AgI precipitates (appearing as white streaks) form along the FIB-milled step edges due to the insolubility of AgI in ethanol.

These results demonstrate that the lateral corrosion depth of the Ag layer in the Ag/Cr-Pt sample is substantially greater than that in the Ag/ITO sample. This confirms that the presence of Cr-Pt accelerates the corrosion of Ag, which is a direct manifestation of galvanic cell functionality.

## 4. Conclusions

This study demonstrates a novel lift-off strategy for the fabrication of LiTaO_3_ metasurfaces, eliminating the reliance on highly toxic etchants. By introducing a thin Ag or Au sacrificial layer (thickness of 30 nm) between the Cr-Pt mask and LiTaO_3_ substrate, complete Cr-Pt mask detachment is achieved by using KI + I_2_ solution—a markedly safer alternative to the aqua regia traditionally required for Pt etching. The key results are summarized as follows:

(1) Rationalized sacrificial layer selection: systematic evaluation identifies Au or Ag as optimal sacrificial layer materials, combining high sputtering yield for efficient FIB patterning with rapid dissolution kinetics in low-toxicity KI + I_2_.
(2) Validated process efficacy: SEM observation reveals well-defined sidewall profiles (10–11°), demonstrating that sacrificial layer incorporation causes no degradation in milling quality. EDS analysis confirms the complete removal of the mask through KI + I_2_ solution. Additionally, AFM results confirm post-lift-off surface roughness comparable to conventional aqua regia processing. The SHG measurements show no notable difference in intensity with the introduction of Ag or Au sacrificial layers, suggesting comparable nonlinear optical performance.
(3) Revealed electrochemical enhancement: the rapid dissolution of Ag—despite its lower intrinsic etch rate—is mechanistically explained by a galvanic coupling effect with the Cr-Pt mask. It has been verified by comparative experiments that lateral corrosion depth of the Ag layer in the Ag/Cr-Pt sample is substantially greater than that in the Ag/ITO sample. This electrochemical acceleration enables highly efficient lift-off of Cr-Pt mask, surpassing the limitations of purely chemical dissolution.

## Figures and Tables

**Table 1:** The properties of metallic mask materials, including sputtering yield under 30 keV Ga^+^ bombardment, compatible etchants, and etchant toxicity [23,24,25].

Mask Materials	Properties		
	Sputtering Yield	Compatible Etchants	Etchant Toxicity/Volatility
Cr	4.7	HNO_3_ + (NH_4_)_2_Ce(NO_3_)_6_	Severe
Ni	7.9	HNO_3_	Severe
Al	3.4	H_3_PO_4_ or NaOH	Strong
Ti	2.3	HF + HNO_3_	Severe
Au	17.0	KI + I_2_	Weak
W	7.4	HF + HNO_3_	Severe
Mo	5.5	HNO_3_ + H_2_SO_4_	Severe
TiN	3.0	HF + HNO_3_	Severe
Co	7.6	HNO_3_	Severe
Ag	13.2	KI + I_2_	Weak

## Data Availability

Data are contained within the article and Supplementary Materials.

## Supplementary Materials

The following supporting information can be downloaded at https://www.mdpi.com/article/10.3390/nano16010059/s1, Figure S1: Illustration of the FIB milling template (2625 × 2625 pixels). The black and white contrast indicates the areas to be retained and milled during the FIB milling process, respectively. Figure S2. SEM image of LiTaO_3_ substrate coated with carbon. The sample is tilted to 52° during SEM observation. Scale bar: 500 nm. Figure S3. Morphological evolution of Au-coated sample before and after etching. (a) Optical image of the sample before etching. (b) Corresponding SEM image. Scale bar: 1 µm. (c) EDS spectrum acquired from the boxed region in (b). (d) Corresponding AFM image before etching. Scale bar: 500 nm. (e) Optical image after etching. (f) Corresponding SEM image after etching. Prior to characterization, the sample is coated with a carbon layer to enhance the surface conductivity. Scale bar: 1 µm. (g) EDS spectrum corresponding to the boxed region in (f). (h) Corresponding AFM image after etching. Scale bar: 500 nm. Figure S4. Compositional evolution of the sample with Cr-Pt mask before and after etching. (a) SEM image of the sample before etching, taken at 52° tilt. Scale bar: 1 µm. (b) Corresponding elemental maps of Cr and Pt from the boxed region in (a). (c) Cross-section SEM image and Pt elemental map outlined in the boxed region. Scale bar: 500 nm. (d) SEM image after etching (sample tilted at 52°). A carbon coating is applied prior to imaging. Scale bar: 1 µm. (e) EDS spectrum collected from the boxed region in (d). (f) Cross-section SEM image and Pt elemental map outlined in the boxed region. Scale bar: 500 nm. White arrows indicate the surface positions of the metasurface units in (c) and (f). Figure S5. Compositional evolution of the sample with an Au/Cr-Pt mask before and after etching. (a) SEM image of the sample before etching, acquired at stage 52° tilt. Scale bar: 1 µm. (b) Elemental maps of Cr and Au corresponding to the boxed region in (a). (c) Cross-section SEM image and Au elemental map of the outlined area. Scale bar: 500 nm. (d) SEM image after etching (52° tilt). The sample is carbon-coated prior to imaging. Scale bar: 1 µm. (e) EDS spectrum acquired from the boxed region in (d). (f) Cross-section SEM image and Au elemental map of the outlined area. Scale bar: 500 nm. White arrows indicate the surface positions of the metasurface units in (c,f). Note that the Au maps in (c,f) show a strong signal from the Pt protective layer due to the spectral overlap between the Au-Mα (2.12 keV) and Pt-Mα (2.05 keV) peaks. Figure S6. Optical images of LiTaO_3_ metasurfaces. (a–c) Images taken immediately after following AFM characterization (before etching) for samples with (a) Cr-Pt, (b) Ag/Cr-Pt, and (c) Au/Cr-Pt masks. (d–f) Corresponding images after etching of the (d) Cr-Pt, (e) Ag/Cr-Pt, and (f) Au/Cr-Pt masks. All scale-bars measure 2 µm. Figure S7. SEM images of LiTaO_3_ metasurface after FIB fabrication in the samples coated with (a) Ag and (b) Au layer. The samples are tilted to 52° to enable lateral observation. All scale-bars measure 1 µm. Figure S8. AFM images of LiTaO_3_ metasurfaces right after FIB fabrication in samples with (a) Cr-Pt mask, (b) Ag/Cr-Pt mask and (c) Au/Cr-Pt mask. Corresponding SEM images of samples with (d) Cr-Pt mask, (e) Ag/Cr-Pt mask and (f) Au/Cr-Pt mask after AFM characterization. The samples are tilted to 52° during SEM observation. All scale-bars measure 1 µm. Figure S9. SEM images of LiTaO_3_ metasurfaces during FIB fabrication in the sample with Au/Cr-Pt mask. The sample is tilted to 52° during SEM observation. All scale-bars measure 1 µm.
