# Supplementary material for: Aqua Regia-Free Removal of Cr-Pt Hard Masks Using Thin Ag or Au Sacrificial Layers for High-Fidelity LiTaO3 Metasurfaces
Source: Nanomaterials (Basel). 2025 Dec 31;16(1):59. doi: 10.3390/nano16010059 (PMC12787872; doi:10.3390/nano16010059)
Supplement: Supplementary file 1 [file nanomaterials-16-00059-s001.zip › nanomaterials-3990700-supplementary.pdf]

Aqua Regia–Free Removal of Cr-Pt Hard Masks Using Thin Ag or Au Sacrificial Layers for High-Fidelity $LiTaO_3$ Metasurfaces

Zhuoqun Wang [1], Yufeng Zang [2], Yuechen Jia [1] and Ning Lu [3]*

[1] School of Physics, State Key Laboratory of Crystal Materials, Shandong University, Jinan, Shandong 250100, China

[2] School of Microelectronics and Communication Engineering, Chongqing University, Chongqing 400044, China

[3] School of Chemistry and Chemical Engineering, Shandong University, Jinan, Shandong 250100, China

* Corresponding author: Ning Lu: nlu@sdu.edu.cn

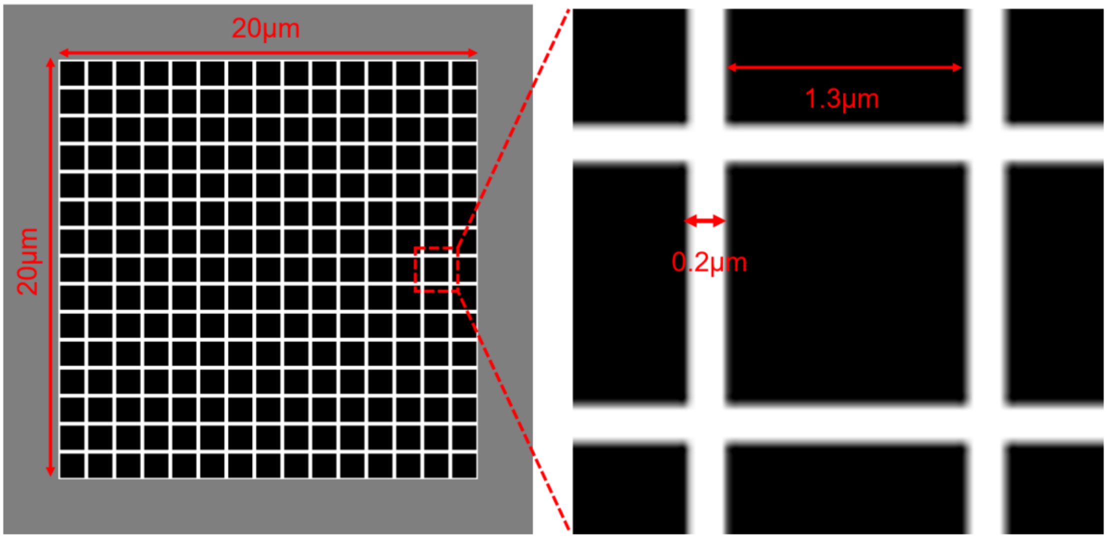

Figure S1. Illustration of the FIB milling template (2625 × 2625 pixels). The black and white contrast indicates the areas to be retained and milled during the FIB milling process, respectively.

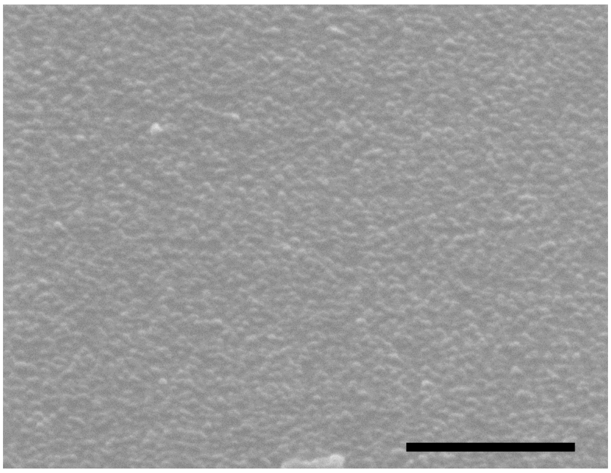

Figure S2. SEM image of $LiTaO_3$ substrate coated with carbon. The sample is tilted to 52° during SEM observation. Scale bar: 500 nm.

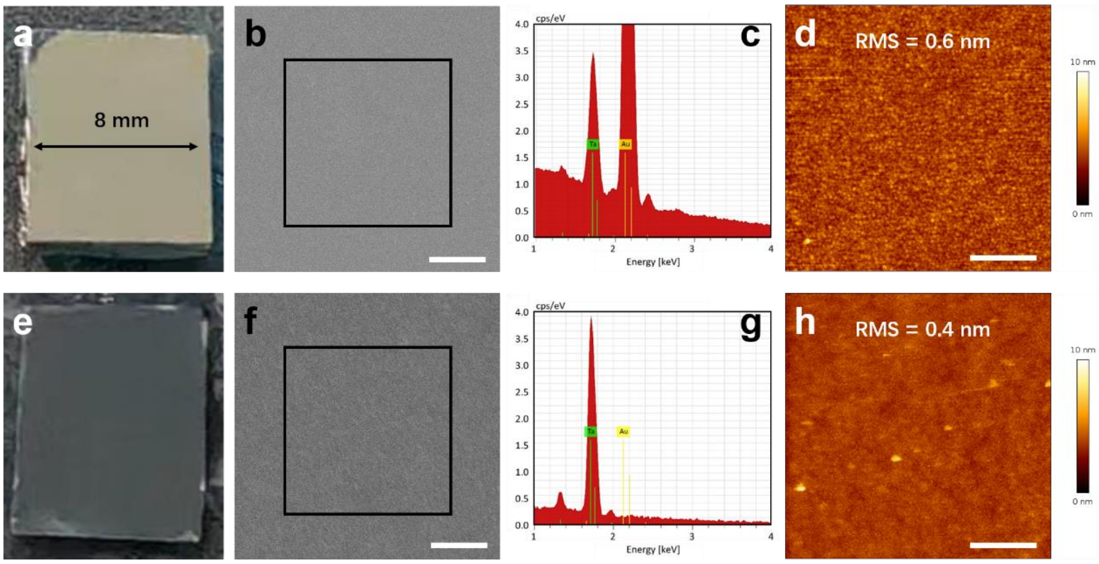

Figure S3. Morphological evolution of Au-coated sample before and after etching. (a) Optical image of the sample before etching. (b) Corresponding SEM image. Scale bar: 1 μm. (c) EDS spectrum acquired from the boxed region in (b). (d) Corresponding AFM image before etching. Scale bar: 500 nm. (e) Optical image after etching. (f) Corresponding SEM image after etching. Prior to characterization, the sample is coated with a carbon layer to enhance the surface conductivity. Scale bar: 1 μm. (g) EDS spectrum corresponding to the boxed region in (f). (h) Corresponding AFM image after etching. Scale bar: 500 nm.

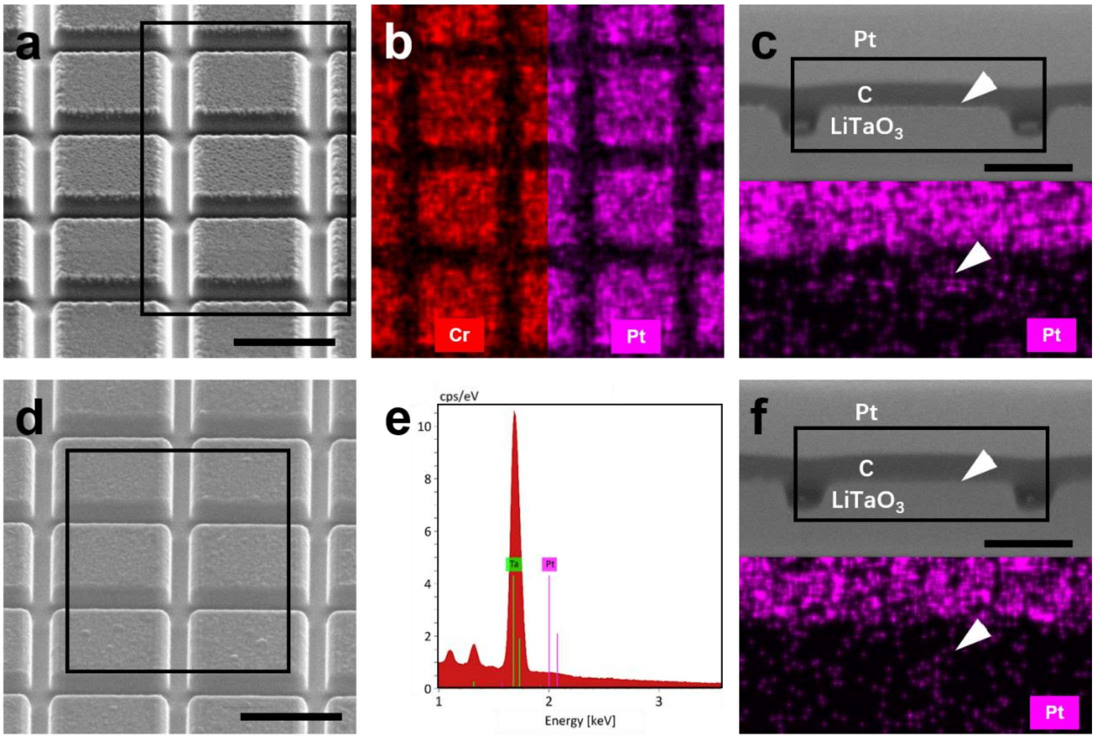

Figure S4. Compositional evolution of the sample with Cr-Pt mask before and after etching. (a) SEM image of the sample before etching, taken at 52° tilt. Scale bar: 1 μm. (b) Corresponding elemental maps of Cr and Pt from the boxed region in (a). (c) Cross-section SEM image and Pt elemental map outlined in the boxed region. Scale bar: 500 nm. (d) SEM image after etching (sample tilted at 52°). A carbon coating is applied prior to imaging. Scale bar: 1 μm. (e) EDS spectrum collected from the boxed region in (d). (f) Cross-section SEM image and Pt elemental map outlined in the boxed region. Scale bar: 500 nm. White arrows indicate the surface positions of the metasurface units in (c) and (f).

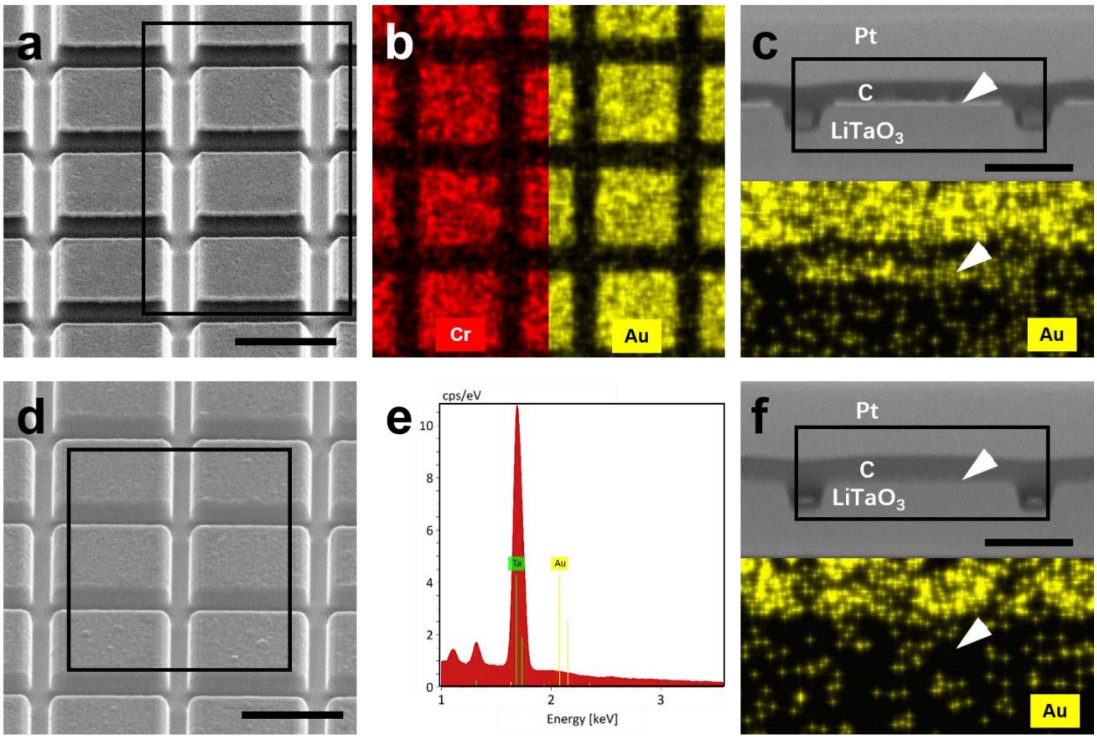

Figure S5. Compositional evolution of the sample with an Au/Cr-Pt mask before and after etching. (a) SEM image of the sample before etching, acquired at stage 52° tilt. Scale bar: 1 μm. (b) Elemental maps of Cr and Au corresponding to the boxed region in (a). (c) Cross-section SEM image and Au elemental map of the outlined area. Scale bar: 500 nm. (d) SEM image after etching (52° tilt). The sample is carbon-coated prior to imaging. Scale bar: 1 μm. (e) EDS spectrum acquired from the boxed region in (d). (f) Cross-section SEM image and Au elemental map of the outlined area. Scale bar: 500 nm. White arrows indicate the surface positions of the metasurface units in (c) and (f). Note that the Au maps in (c) and (f) show a strong signal from the Pt protective layer due to the spectral overlap between the Au-Mα (2.12 keV) and Pt-Mα (2.05 keV) peaks.

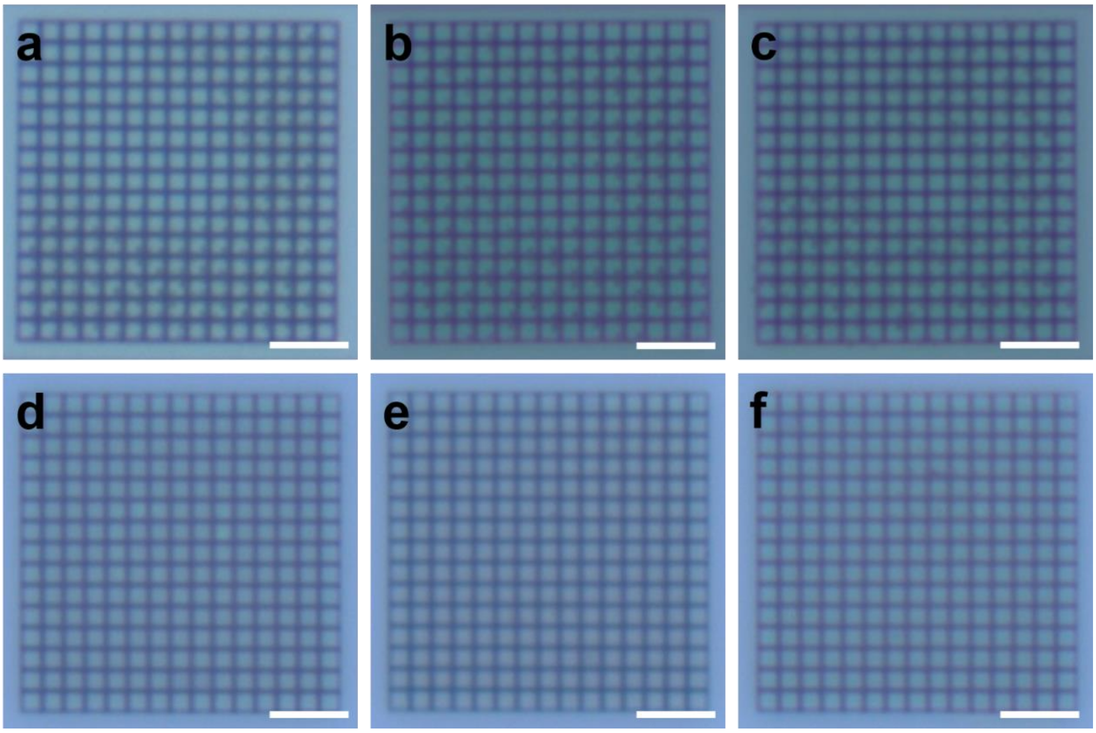

Figure S6. Optical images of $LiTaO_3$ metasurfaces. (a–c) Images taken immediately after following AFM characterization (before etching) for samples with (a) Cr-Pt, (b) Ag/Cr-Pt, and (c) Au/Cr-Pt masks. (d–f) Corresponding images after etching of the (d) Cr-Pt, (e) Ag/Cr-Pt, and (f) Au/Cr-Pt masks. All scale-bars measure 2 μm.

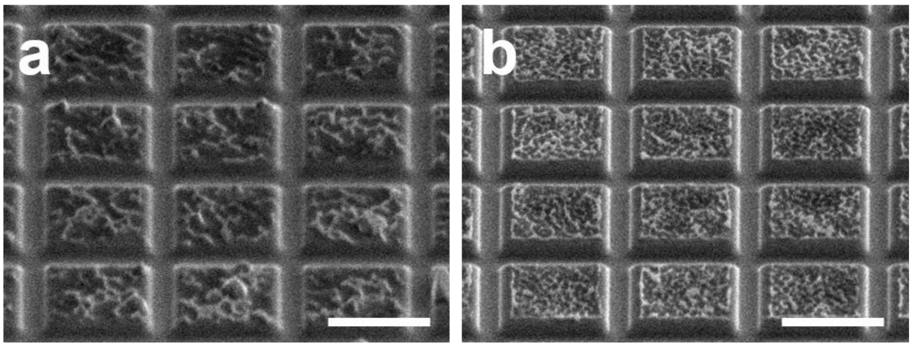

Figure S7. SEM images of $LiTaO_3$ metasurface after FIB fabrication in the samples coated with (a) Ag and (b) Au layer. The samples are tilted to 52° to enable lateral observation. All scale-bars measure 1 μm.

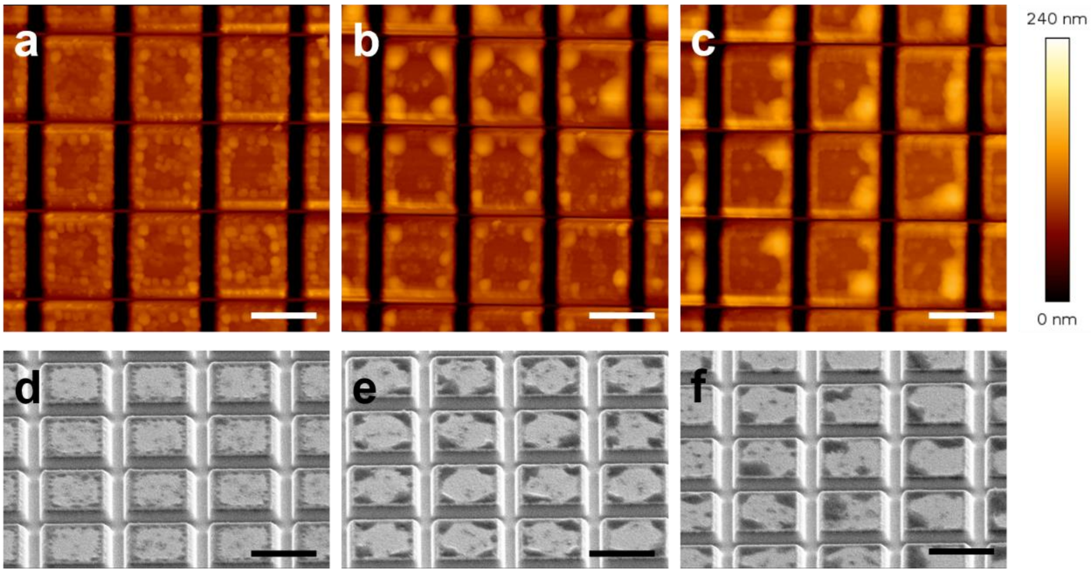

Figure S8. AFM images of $LiTaO_3$ metasurfaces right after FIB fabrication in samples with (a) Cr-Pt mask, (b) Ag/Cr-Pt mask and (c) Au/Cr-Pt mask. Corresponding SEM images of samples with (d) Cr-Pt mask, (e) Ag/Cr-Pt mask and (f) Au/Cr-Pt mask after AFM characterization. The samples are tilted to 52° during SEM observation. All scale-bars measure 1 μm.

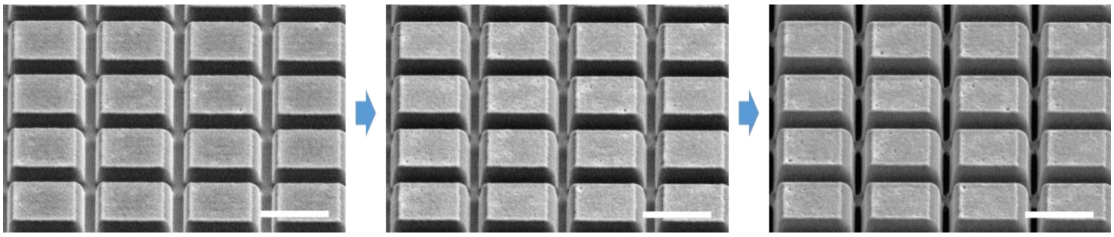

Figure S9. SEM images of $LiTaO_3$ metasurfaces during FIB fabrication in the sample with Au/Cr-Pt mask. The sample is tilted to 52° during SEM observation. All scale-bars measure 1 μm.
